# Microbial Ecology from the Himalayan Cryosphere Perspective

**DOI:** 10.3390/microorganisms8020257

**Published:** 2020-02-14

**Authors:** Kusum Dhakar, Anita Pandey

**Affiliations:** 1 Newe Ya’ar Research Center, Agricultural Research Organization, Ramat Yishay 30095, Israel; kusumdhakar@gmail.com; 2 Department of Biotechnology, Graphic Era (Deemed to be University), Bell Road, Clement Town, Dehradun 248002, India

**Keywords:** cryosphere, Himalaya, microbial communities, cold tolerant microorganisms

## Abstract

Cold-adapted microorganisms represent a large fraction of biomass on Earth because of the dominance of low-temperature environments. Extreme cold environments are mainly dependent on microbial activities because this climate restricts higher plants and animals. Himalaya is one of the most important cold environments on Earth as it shares climatic similarities with the polar regions. It includes a wide range of ecosystems, from temperate to extreme cold, distributed along the higher altitudes. These regions are characterized as stressful environments because of the heavy exposure to harmful rays, scarcity of nutrition, and freezing conditions. The microorganisms that colonize these regions are recognized as cold-tolerant (psychrotolerants) or/and cold-loving (psychrophiles) microorganisms. These microorganisms possess several structural and functional adaptations in order to perform normal life processes under the stressful low-temperature environments. Their biological activities maintain the nutrient flux in the environment and contribute to the global biogeochemical cycles. Limited culture-dependent and culture-independent studies have revealed their diversity in community structure and functional potential. Apart from the ecological importance, these microorganisms have been recognized as source of cold-active enzymes and novel bioactive compounds of industrial and biotechnological importance. Being an important part of the cryosphere, Himalaya needs to be explored at different dimensions related to the life of the inhabiting extremophiles. The present review discusses the distinct facts associated with microbial ecology from the Himalayan cryosphere perspective.

## 1. Introduction

### 1.1. Himalaya: Geographic Importance and Its Uniqueness

**Figure 1:**
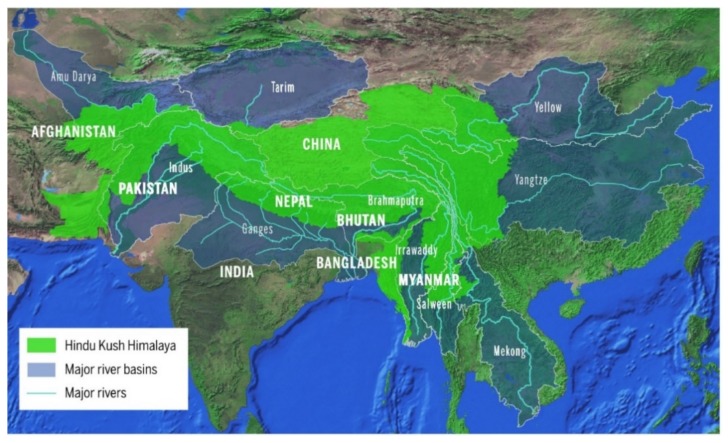
Geographical distribution of Himalayan ranges (Source: ICIMOD (International Centre for Integrated Mountain Development), Nepal).

Our planet is a cold planet because the majority experiences temperatures below 15 °C throughout the year. Himalaya is one of the geographic locations that is recognized as a unique low-temperature environment since it holds the highest glacial coverage after the polar regions [1]. Dimensions of Himalaya are vast (>2000 km in plains and height up to >7000 m asl) and distributed widely in the Asian continent, and they control the weather conditions of the surrounding international landmarks/regions (Figure 1). Various ecosystems are found in the huge covering area of these mountain ranges. Himalaya is recognized with highly diverse regions in terms of geographical and biological aspects. It includes evergreen forests, lakes, hot springs, cold deserts, glaciers, grass lands, and so forth [2]. Further, the Himalayan mountain ranges offer a huge natural resource in the regions and support several rivers as source of water in the continent [3]. These distinct geographic regions have climatic dissimilarities that make the Himalayan region a hot spot of biodiversity. From foothills to the top, the mountain ranges are recorded to have unique and highly different biological components.

Besides the polar region, Himalaya possesses the highest altitude (approx. 8000 m asl) and represents similar environmental conditions to the other extreme colder regions on Earth. Its widely known low-temperature regions restrict the growth of higher plants and animals. The normal life in such locations is challenging; therefore, very few animals or plants can be seen surviving in these regions [4]. The extreme colder environments possess several other climatic factors that generate stress for life (e.g., high exposure to harmful radiation, low nutrient and water availability, desiccation, etc.) [5,6]. The extreme colder regions are mainly colonized by cold-adapted microorganisms only, “pyschrophiles/psychrotolerants” (cold-loving microorganisms) [7]. Such cold-loving microorganisms are known to have several modifications at cellular and biochemical levels to alleviate the effect of low temperatures and other associated stress conditions. These extreme low temperatures are dependent on these environment-specific microorganisms for their several ecological processes [8]. In the socio-economic perspective, Himalayan ranges serve as home to the population living in it. The mountains provide resources to the people where agriculture is one of the most important tasks that feeds the settlements and maintains the energy network in the system [9]. In the present review, we discuss the cold-adapted microorganisms in view of their contributions to the low-temperature environments of Himalaya. 

### 1.2. Adaptations: Flexibility of Metabolic Processes Under Cold Conditions

**Figure 2:**
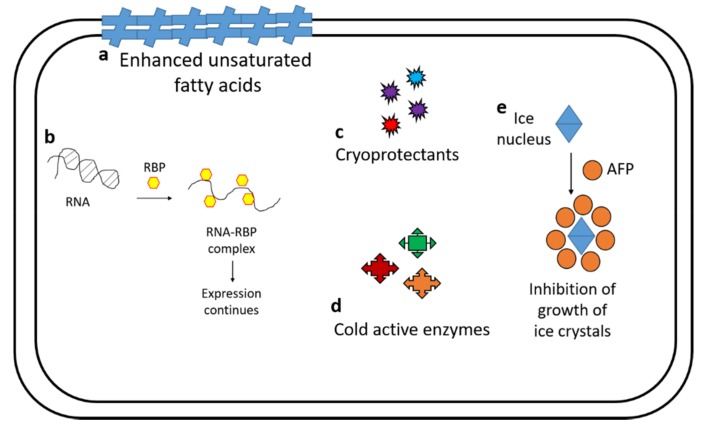
To maintain cellular integrity and functionality, cold-loving microbial cells possess some extraordinary features—**a**: increase in unsaturated fatty acids against desiccation, **b**: RNA binding proteins (RBP) help to retain the RNA primary structure to support expression, **c**, **d**: Accumulation of cryoprotectants and functioning of cold-active enzymes support homeostasis and growth in stressed environments, **e**: inhibition of ice crystal formation protects the cell from damage at low temperatures.

Cold-adapted microorganisms have evolved metabolic processes to cope with the adverse effects of low temperatures (Figure 2). Freezing temperatures cause cell damage due to desiccation and affects physiological reactions adversely. The cold-adapted microorganisms have evolved strategies at biochemical and molecular levels to withstand against the extreme low temperatures [10]. The cold-adapted microorganisms change their lipid composition (increased level of unsaturated fatty acids) to maintain cell membrane integrity under low-temperature environments. A recent study suggested that the microorganisms switch among different metabolic pathways in response to low temperature to obtain energy [11]. The accumulation of cryoprotectants such as trehalose, glycerol, mannitol, and so forth is responsible for maintaining homeostasis in the cell [12]. The cold-active enzymes have conformational flexibility to balance the low kinetic energy with high catalytic efficiency and increased turnover number under low-temperature environments [13]. The low-temperature effect is also associated with oxidative stress, which is overcome by antioxidants to retain a normal cellular environment [14]. Presence of cold shock proteins is reported from different groups such as prokaryotes, animals, and plants. In prokaryotic systems, up-regulation of chaperons (*csp*, *ctr*, *rbps*, etc.) has been studied to support growth under extreme low temperature [15,16]. These RNA-binding proteins (generally glycine-rich RNA binding domains) possess the ability to bind the RNA to destabilize the secondary structures and help in the proper functioning of the biochemical processes [17]. Antifreeze proteins (AFPs)/Ice binding proteins are one of the most important adaptations reported in the organisms (animals, plants, and microorganisms) inhabiting extreme low temperatures. They are the ice binding proteins and help to facilitate microbial growth by inhibiting the formation of ice crystals under extreme low temperatures [18,19]. Except for extreme temperatures, these environments are characterized with oligotrophy, desiccation, radiation, and so forth. The thriving microbes are capable of producing pigments to alleviate the effect of radiation and evolve to tolerate high salt concentration to avoid the adverse effect of desiccation. The pH tolerance of these microorganisms possesses the interesting fact that the microbial component associated with these cold regions has been found to tolerate a wide pH range (whereas the habitat is normal in terms of pH 5–7). Molecular investigations need to be done on the mechanisms behind this tolerance as it could be an important characteristic for novel biotechnological products [20]. Apart from these adaptations, the presence of pigments in cold-adapted microorganisms is recognized as an important adaptation against harmful radiation [21]. Recently, the production of various pigments in the microbiota of Himalayan soils has been reported and is likely to have a role in successional processes [22].

Investigations on the cold-adapted microorganisms have been focused on the most extreme low-temperature environments only. However, the climatic conditions in the Himalaya are somewhat different. Because of the relatively broad spectrum of environmental conditions that prevail, the Himalayan region favors colonization of the “tolerants” in comparison to “philes”. While growth of the latter is restricted to specific climatic conditions, the tolerants, being more diverse, can survive and colonize under a range of external factors. Investigations on the cellular and metabolic processes of these cold-tolerant microorganisms can help in revealing the connection between obligates (philes) and generalists (tolerants). The life processes associated with tolerants are more complicated and important in environmental and industrial perspectives. 

## 2. Microbial Diversity: Compositional and Functional Spectrum 

Himalaya is one of the regions representing low-temperature environments (also considered as the “third pole” because of its environmental similarity to the polar regions) on Earth [23]. Earlier, it was thought that these extreme colder regions were sterile and did not contain any life, but the advancement in science and technology revealed a huge and complicated world of these tiny warriors inhabiting in the extreme low-temperature environments (glaciers, snow, ice, etc.) and playing key roles in the environmental processes [24]. The cryosphere, in general, has become a major fraction of global ecology. The fast upgradation in technology stretched the resolution for microbial ecology and revealed the colonization of all the three domains of life in colder regions. The upcoming techniques are likely to provide more information about the dead and the live microbial communities and their activities. Omics approaches have already brought huge information at the molecular level with respect to taxonomic aspects of the microbial communities, enhancing our understanding on microbial interactions in specific niches [25,26]. Similarly, the microbial diversity of an important fraction of the cryosphere, the Himalayas, is being investigated by several research groups with various traditional and high-throughput methods [27].

The existing studies from different regions of Himalaya show the complicated composition of microbial communities [28,29,30,31,32] and diverse metabolic potential [27,33,34] through various culture-dependent and culture-independent methods. In the last two decades, a lot of information associated with the microbial communities of Himalaya has been generated. In the early stages, prokaryotes were highly targeted among the microbial components because of their high involvement in diverse ecological processes [35]. Only few studies are dedicated to the distribution of fungi in the Himalayan region, indicating the significantly high abundance of Ascomycota [5,36,37]. The investigations on these inhabiting micro-eukaryotes are now increasing continuously [29,38,39] because of their high bioprospection values in agriculture and other biotechnology-based industries [27].

Towards bacterial communities, Gangwar et al. [40] reported high dominance of Proteobacteria in culture-dependent and culture-independent studies from the soil of different altitudes of Himalaya, with a reduction in the bacterial genera along the increasing altitude. Even the change in bacterial diversity from subalpine to subtropical regions was observed through TTGE (temporal temperature gradient gel electrophoresis) [41]. The presence of temperature-sensitive and tolerant bacterial isolates has been reported from the glacial sites of Mount Everest, which is an indication of the diversity richness even in the most extreme and harsh regions [42]. Except this, several known glaciers of Himalaya (such as Changme Khangpu, Hamta, Pindari, Kafni, and Roopkund) have been investigated for their bacterial communities through 16S rRNA gene amplicon sequencing along with the traditional approach [43,44,45,46]. In fact, these regions have also been reported as a source of novel species [47,48,49]. The phototrophic microbial communities of the Ladakh region were assessed and reported with a diverse composition of cyanobacteria and eukaryotic algae across the altitude between 3700–5900 m asl [50]. Diversity of cold-adapted bacilli has also been investigated in subglacial lakes located in Indian Himalaya following the amplified ribosomal DNA restriction analysis (ARDRA) method. Further, the bacterial diversity has been recognized for its potential in the production of cold-active enzymes [51]. In addition, records from similar regions revealed the presence of sulfate-reducing and anaerobic bacteria through the phosphor lipid fatty acids (PLFA) analysis [52]. Metagenomic investigations of Pangong Lake located in Himalaya revealed a complex composition of microbiota along with the dominance of bacteria (where Proteobacteria >50%). The microbial communities were found to be mainly associated with the biogeochemical cycle of carbon and nitrogen [53]. In a recent study, the culture-dependent bacterial diversity (high abundance of Firmicutes and Proteobacteria) of the glacial sites of Himalaya has been reported, which showed the importance of Matrix-Assisted Laser Desorption/Ionization–Time Of Flight (MALDI-TOF) mass spectrometry along with nucleic acid based methods, in the identification of cold-tolerant bacterial strains [54].

The abundance of Actinobacteria, Proteobacteria, and Firmicutes in the Himalayan region was similar to the bacterial genera found in the other colder regions (such as Antarctic and Arctic) [46,55]. Because of the environmental similarities between polar areas, microbial components have been found similar, to some extent, in both the colder regions. However, the barren soils of dry valleys located in Himalaya are found to have a rich photoautotrophic microbial diversity in the low-mineral soils, similar to Antarctic dry valleys [56,57]. Yet, more investigations on the molecular level are awaited. These valleys are characterized by mineral-limited soils at high elevation along with extreme temperature fluctuations at a large scale across the freezing temperature. The associated microbial diversity has shown similarity to the Antarctic arid soils and other high-altitude regions, indicating the close relationship of their climatic conditions. Such comparative studies have an advantage towards the distribution of biogeochemical processes across the globe and to understand the analog extremes of Mars, which is characterized by low-mineral soils with extreme cold [57,58]. 

The soils at high altitude at the retreating of glaciers have become an important ecosystem in the climate change perspective as these barren soils are transforming due to colonization of various new microbial communities. These communities consist of diverse microorganisms, heterotrophs to chemotrophs, that contribute significantly to the biogeochemical cycles in the developing stage of soils. The abundance of cyanobacteria indicates their key role in increasing soil carbon and nitrogen content through other environmental processes [59]. Similar results on the significant contribution of the heterotrophs in the carbon and nitrogen inputs have been reported from the debris covering glaciers [60]. The periglacial soils from different regions (Rocky, Andes, and Himalayas) of the world revealed the unity in the inhabiting fungal component. It has been widely recognized that soils in colder regions are dominated by Ascomycota and Basidiomycota. In contrast, high elevations of mountains that experience high snow falls have been found to be abundant in Chytridiomycota, the zoosporic fungi [61,62]. Probably, the continuous freeze-and-thaw cycles and moisture content lead to the dominance of such lower fungi that are linked between the aquatic and terrestrial ecosystems. 

The Tibetan Plateau represents the world’s highest plateau that contains glaciers with extremely cold and unique habitats for colonization of microorganisms in the ice sheets [32]. Probably, aeolian activities contribute towards the microbial transport. An extensive bacterial diversity (15 genera approximately) with prevalence of Proteobacteria has been reported through the molecular methods [63,64]. The autotrophic microbial communities (consisting of Rhizobiales, Burkholderiales, and Actinomycetales) are mainly responsible for the biological processes and nutrient cycling (including carbon and nitrogen) in the semiarid/arid ecological conditions with restricted vegetation [65]. The wetlands located in the region (Tibetan Plateau) are considered as a significant contributor to the biogeochemical cycling of carbon and other nutrients. However, they have been less explored for their microbial communities because of their heterogenous biogeography [66]. 

Besides glaciers, snow, lakes, or sediments from the barren lands, unique sites with high temperatures (Manikaran, Soldhar, and Ringigad hot springs) have also been located in the Himalayas [67]. Even in the presence of very high temperatures (>40 °C), the complexity of microbial communities is maintained. These hot springs have been reported to possess a high diversity of hyperthermophiles and thermophilic bacteria [68,69,70,71,72,73]. The microbial interactions and the flow of flux was also assessed through metagenome analysis [74,75]. However, advanced investigations on these thermophilic microbial communities with respect to their role in biogeochemical cycles and adaptations are awaited. 

Apart from the extensive molecular studies of microbial communities, there are some studies available that provide the status of the microbial biomass in the soil affected by the climatic conditions/anthropogenic activities [76,77]. Forest fires are one of the main anthropogenic activities that exerts a negative effect to the beneficial microbiota [78]. 

Although a plethora of information on the microbial communities associated with the colder environments is available, understanding the ecological processes and the interactions among the microbial communities is still in its infancy. As per the records, bacterial groups are frequently targeted, whereas information of the fungal component remains scarce. Further, apart from high-throughput methods used in ecological studies, environmental factors still restrict the understanding on microbial interactions effectively. More investigations are required targeting functional attributes that can help us to understand the complex network of the energy flow in such microbial communities. It is widely known that the microbial communities of the low-temperature environments are highly sensitive to temperature changes; therefore, they can serve as indicators of climate change. Out of all these important issues, the cold-adapted microbial communities require more investigation regarding their isolation, characterization, preservation, and careful utilization for various industries. 

## 3. Bioprospection of Cold-Tolerant Microbes: Versatile and Promising Candidates for Various Applications

The cold-adaptive microorganisms are recognized as potential source of cold-active enzymes and bioactive compounds [7]. In the present scenario, the industries are diverting towards green chemistry to use enzyme-based reactions rather than involve chemicals such as in the textile industries [79]. Working with enzymes has several advantages, such as less interference on byproducts, reduced energy consumption with negligible pollution, and no compromise of product quality. The cold-active enzymes have shown low activation enthalpy and flexibility in structures. Such thermodynamic parameters help these cold-active enzymes to perform catalytic actions under the low kinetic energy of cold environments [80]. Lipase, cellulase, amylase, invertase, protease, and lignin-degrading enzymes, produced by cold-adaptive microorganisms, have various applications in detergent, food, the pharmaceutical industry, and so forth [81]. Antifreeze proteins from cold-adaptive organisms have also been recognized for their important physiological and biotechnological contributions. They are the group of proteins that control the shape and size of ice crystals and allow the biochemical activities below freezing temperatures. The production of AFPs from different organisms is the result of convergent evolution through the pressure of extreme cold temperature. The AFPs are reported for other industrial applications too [82,83].

The microorganisms colonizing the extreme colder regions are adapted to cope with various stress conditions (limited nutrient availability, radiations, desiccation, etc.) and produce novel metabolites that are absent in the mesophiles. These metabolites have significant importance in various industries such as food, textiles, therapeutics, and so forth [84,85]. Bioactive compounds related to antimicrobial and immunosuppressive activities, for example, have also been isolated from the cold environment microorganisms [86]. 

The low-temperature environments of Himalaya are seen as a promising source for novel products, bioactive compounds, and other industrially relevant substances/compounds [22,27,87]. Metagenomic investigations reveal the functional potential of the inhabiting soil microbial communities and allow to study the metabolic pathways and interactions associated with the uncultured biological components also [88]. For example, cold-active enzymes (amylases, endocellulases) were obtained from the clone library and are highly active at low temperatures [89,90]. These low-temperature active products can be used in industries to reduce energy consumption. Apart from this, the multicopper enzymes, well known as versatile and efficient tools against a range of complex compounds, are highly distributed in the region. Potential to produce laccase enzyme from the cold-adapted microorganisms of glacial sites and the hot springs of Himalaya have been reported through culture-dependent and culture-independent methods [91,92]. The cold-adapted microorganisms of Himalayan soils have also been reported as an abundant source of diverse cold-active lipases [93,94] that have importance in bioremediation, food industries, and cleaning industries [95]. The Himalayan soils are found to possess high diversity of bacteria that produce carbonic anhydrase, which is an important candidate for investigations related to carbon sequestration [96]. Among the microorganisms, cold-adapted yeasts are recognized as an important group for their survival strategies and biotechnological benefits [97]. There are some records available on these single-cell eukaryotes from Himalaya; however, more investigations are awaited for their ecological contributions [98]. 

**Figure 3:**
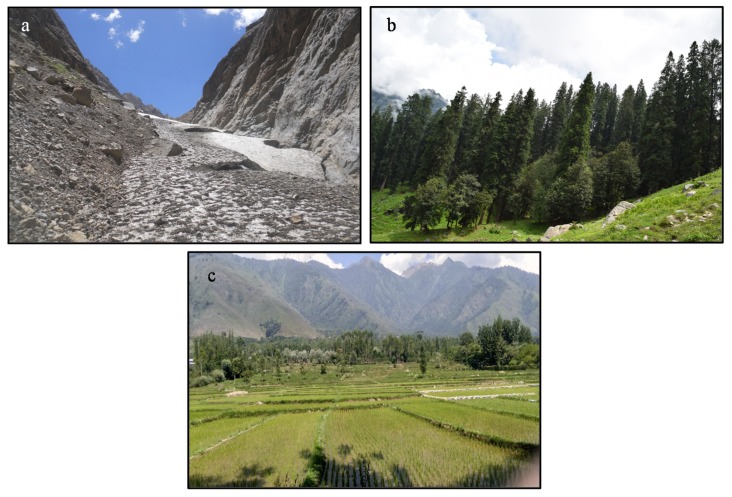
Ecological processes regulated by cold-adapted microorganisms in different ecosystems. (**a**) Biogeochemical cycles in the barren lands of glacial regions (>3500 m asl). (**b**) Nutrient cycles/energy flow in the high-altitude forests. (**c**) Agriculture system under the low-temperature regions of mountains (Photo credit: Khashti Dasila).

Not only the biotechnological benefits but also the inhabiting cold loving microorganisms contribute to several ecological processes in different ecosystems located in Himalaya (Figure 3). In the environmental importance, biodegradation and agriculture (such as plant growth promoting activities and biocontrol) under the colder regions are well supported by the inhabiting microbes [99,100]. These microorganisms have the capability to degrade a range of compounds that have been identified as strong agents in bioremediation. They are also involved in the carbon cycle and contribute in the energy flux in the environment. *Pseudomonas*, *Acinetobacter*, *Rhodococcus*, *Bacillus*, and *Sphingobium* have been found to contribute significantly to the degradation of complex hydrocarbons (such as petroleum products) under low-temperature environments [101,102,103,104]. The presence of a particular hydrocarbon compound also affects the environment on the ecological ground. For example, a colder area (China–Russia) contaminated with petroleum revealed the microbial shifts and the abundance of efficient degraders (endemic communities) [105].

**Figure 4:**
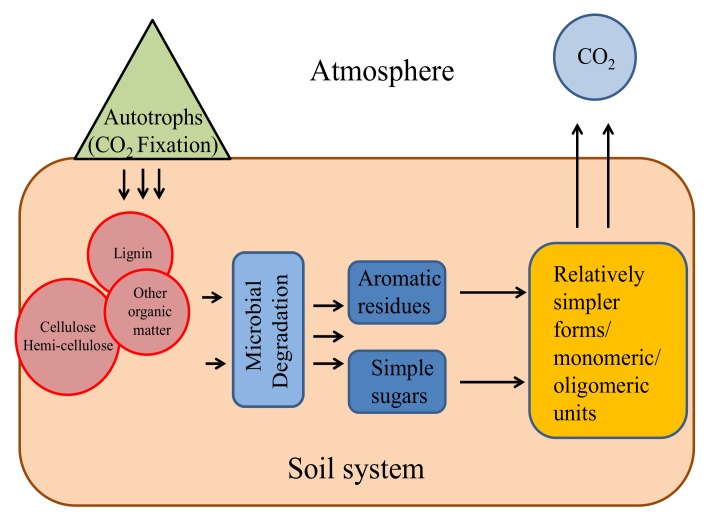
Conversion of different forms of carbon through microbial activities under the biodegradation process.

The biodegradation process in low-temperature environments is very slow and restricted. Organic carbon is sequestered and accumulates in these environments (in the form of lignocellulosic material). These cold-adapted bacteria were also found to possess different hydrolytic enzymatic activities indicating their contribution in the cycling of nutrients including carbon [34,40,106]. Studies from Indian Himalaya have proved that the associated psychrotolerant spp. (bacteria and fungi) have the potential to produce lignin-degrading enzymes at a wide range of temperature and pH [91,107] (Figure 4). 

Apart from degradation, the inhabiting microbial communities have shown their immense contribution to improve the agriculture in low to extreme low temperature environments of Himalaya. These psychrotrophic/psychrophilic microorganisms have been reported for their potential in plant growth promotion activities. They are found to possess activities, such as phosphate solubilization, indole acetic acid production, and siderophore secretion, that are useful in agriculture under low-temperature environments [52,108]. In the recent two decades, several researchers have published the potential utilization of microbial strains from Indian Himalaya. *Rhodococcus*, *Pseudomonas*, *Bacillus*, *Serratia*, and Ascomycetous fungi (*Trichoderma*, *Aspergillus*, *Penicillium*) have been recognized as promising candidates in agriculture and forestry [100,109,110,111,112], but they need further attention for obtaining a form of valuable product. Recently, a strain of *Stenotrophomonas* with multiple plant growth promoting activities and possibilities to utilize as biofertilizer has been reported [113]. In last few years, a range of endophytes have been isolated and screened for their importance in agriculture under the low-temperature environments of Himalaya [114,115]; except for this, they have also been recognized as a good source of antimicrobial compounds [116]. Towards ecological aspects, the specific dark septate endophytes have been also investigated from the high altitude of Himalaya, and they await their recognition for their role as indicators of the changing climate under the mountain ecosystem [117]. By considering the ecological and biotechnological importance of the endophytes, it is important to investigate this group effectively because they are likely to be the important players that help in the nutrient flux between soil and plants (especially in the nutrient-poor soils of high altitudes of Himalaya).

Additional information on the taxonomic and functional aspects of microbial communities of Himalayan regions is provided in Table 1.

## 4. Knowledge Gaps and Future Prospects

So far, we have discussed the importance of Himalaya as a fraction of the cryosphere, which contributes significantly to the global ecology. Although the microbial diversity of Himalaya has been explored by various research groups, still the information related to the microbial ecology of this unique region is scarce. Most researchers have focused on the taxonomic aspects, while the microbial interactions and functional ecology remain neglected. Further, the diversity or the composition of the most important micro-eukaryotes in the Himalayan region is not targeted well, neither with culture-dependent nor with culture-independent methods. Since Himalaya shares climatic similarities with the polar regions, information on the microbial composition and the metabolic activities needs to be investigated so that it can be compared to the extreme colder regions to identify microbial resources (e.g., Antarctic and Arctic investigated for novel antibiotics, degraders, etc.). The gene pool of several unique sites of Himalaya (most of the glaciers, rock-associated microbial communities) is still untouched. The gene pool is precious because of its wide tolerance against a range of environmental conditions, such as high-temperature fluctuation and precipitation in lush green forests to barren cold deserts.

Majority of these studies report high abundance of extremotolerant species that might be due to a broad fluctuation in temperature (extreme low to normal 30–40 °C) of various regions of Himalaya. This broad range of temperature change allows the mesophiles and the tolerants to grow actively in the environment. This could be one the reasons for the distinct metabolic ability of Himalayan microorganisms from the microbial communities of the Antarctic region [31].

Besides, in the present scenario of global warming, these cold environments are being recognized as highly affected regions because of their sensitivity towards temperature change. While most of the studies provide information on the shifts in microbial communities in various environments of the polar regions, the ecology of the Himalayas still needs focused attention. Very rare or negligible information is on record about the transformation of the microbial activities under the changing environmental conditions. The change in green cover and glacier retreat are documented for Arctic and Antarctic, but no such authentic information is provided in the context of Himalaya.

It will be important to explore the extreme cold regions of Himalaya to enhance our understanding on the metabolic activities in the low-temperature environments. This would help us to identify ecological problems associated with the mountain system, leading to the efficient use of natural resources along with the preservation of the unique biodiversity, the microbial diversity in particular, of Himalaya. 

## Figures and Tables

**Table 1:** Taxonomic and functional information associated with the microbial communities of Himalayan region.

Serial. No.	Location	Microorganisms	Report	References
1	Northwestern Himalaya, India	Fungi	Plant growth promoting activities of *Penicillium* spp.	[118]
2	Uttarakhand, India	Bacteria	Antagonistic activity	[119]
3	Uttarakhand, India	Bacteria	Thermophiles from hot springs	[120]
4	Uttarakhand, India	Bacteria	Plant growth promoting activities	[121]
5	Kargil, India	Bacteria	Cold-active amylases (culture independent)	[90]
6	Annapurna and Sagarmatha region, Nepal	Microbial communities	Microbial biomass and associated ecological processes	[122]
7	Langtang Valley, Nepal	Bacteria	Bacterial diversity of glaciers (culture independent)	[123]
8	North-Central Nepal	Phototrophs	Diversity of cyanobacteria and Chlorophyceae (culture independent)	[57]
9	Arunachal Pradesh, India	Fungi	Antimicrobial activity	[124]
10	Northeast India	Bacteria	Bacterial diversity (culture dependent)	[125]
11	Uttarakhand, India	Microbial communities	Comparative study of soil microbial communities by PLFA (culture independent)	[126]
12	Northwestern Himalayas, India	Bacteria	Plant growth promoting activities of *Pseudomonas*	[127]
13	Khumbu Valley, Nepal	Micro-eukaryotes	Planktonic diversity (culture independent)	[128]
14	Uttarakhand, India	Bacteria	Plant growth promoting activities	[111]
15	Himachal Pradesh, India	Bacteria	Plant growth promoting activities (culture dependent)	[52]
16	Ladakh, India	Bacteria	Cyanobacterial communities	[129]
17	Uttarakhand, India	Functional aspect of bacterial communities	Distribution of *nifH* gene through metagenomics	[130]
18	Ladakh, India	Microbial communities	Metagenomics based study of Pangong lake water	[53]
19	Indian Himalaya (Himachal Pradesh and Sikkim)	Bacteria	Potential of hot spring inhabiting bacteria to produce thermostable enzymes (culture dependent)	[72]
20	Khumbu Valley, Nepal	Bacteria	Bacterial diversity associated to snow (culture independent)	[131]
21	Uttarakhand, India	Microbial communities	Microbial biomass associated to the forests and their relation to ecological processes	[76]
22	Ladakh, India	Bacteria	Metagenomics	[132]
23	Himachal Pradesh, India	Bacteria	Bioplastic-producing bacterial communities (culture independent)	[133]
24	Sikkim, India	Bacteria	Contribution of thermophiles in nitrogen and sulfur cycle	[134]
25	Indian Himalaya	Bacteria and fungi	Degradation potential of cryoconites inhabiting microbial communities (culture dependent)	[31]
26	Himachal Pradesh, India	Bacterial and Archaeal viruses	Abundance of viruses in the Manikaran hot spring through metagenomics	[74]
27	Sikkim, India	Bacteria	Culture-dependent diversity	[44]
28	Northwestern Himalaya, India	Cyanobacteria	Diversity of cyanobacteria in hot spring (culture dependent)	[135]
29	Himachal Pradesh, India	Bacteria	Cellulolytic potential	[33]
30	Himachal Pradesh, India	Bacteria	Potential to degrade enzymes from bacteria colonizing hot spring	[136]
31	Himachal Pradesh and Uttarakhand, India	Bacteria	CO_2_ mineralization through carbonic anhydrase	[96]
32	Uttarakhand, India	Bacteria	Potential of cold-tolerant bacteria in hill agriculture (culture independent)	[29]
33	Himachal Pradesh, India	Bacteria and Archaea	Taxonomic and functional potential of hyperthermophiles through metagenomics	[75]
34	Sikkim, India	Bacteria	Culture-independent diversity	[30]
35	Uttarakhand, India	Actinomycetes	Antimicrobial activity	[137]
